# Evaluation of fluxon synapse device based on superconducting loops for energy efficient neuromorphic computing

**DOI:** 10.3389/fnins.2025.1511371

**Published:** 2025-02-14

**Authors:** Ashwani Kumar, Uday S. Goteti, Ertugrul Cubukcu, Robert C. Dynes, Duygu Kuzum

**Affiliations:** ^1^Department of Electrical and Computer Engineering, University of California San Diego, San Diego, CA, United States; ^2^Department of Physics, University of California San Diego, San Diego, CA, United States; ^3^Department of Chemical and Nano Engineering, University of California San Diego, San Diego, CA, United States

**Keywords:** neuromorphic computing, superconducting loops, Josephson junctions, deep learning, image classification, energy efficient hardware

## Abstract

With Moore’s law nearing its end due to the physical scaling limitations of CMOS technology, alternative computing approaches have gained considerable attention as ways to improve computing performance. Here, we evaluate performance prospects of a new approach based on disordered superconducting loops with Josephson-junctions for energy efficient neuromorphic computing. Synaptic weights can be stored as internal trapped fluxon states of three superconducting loops connected with multiple Josephson-junctions (JJ) and modulated by input signals applied in the form of discrete fluxons (quantized flux) in a controlled manner. The stable trapped fluxon state directs the incoming flux through different pathways with the flow statistics representing different synaptic weights. We explore implementation of matrix–vector-multiplication (MVM) operations using arrays of these fluxon synapse devices. We investigate the energy efficiency of online-learning of MNIST dataset. Our results suggest that the fluxon synapse array can provide ~100× reduction in energy consumption compared to other state-of-the-art synaptic devices. This work presents a proof-of-concept that will pave the way for development of high-speed and highly energy efficient neuromorphic computing systems based on superconducting materials.

## 1 Introduction

In the era of artificial intelligence (AI), artificial neural networks (ANNs) are at the forefront of the modern computing paradigms used in a wide range of applications, including image and video classification, pattern recognition, natural language processing (NLP), autonomous vehicles, robotics, gaming, virtual reality, and bioinformatics (Heaton, 2018; LeCun et al., 2015; O'Kelly et al., 2020; Schmidhuber, 2015; Vamathevan et al., 2019; Vaswani et al., 2017) The complexity and scaling requirement of targeted applications push AI models towards more complex and larger architectures. This results in significant increase in the overall energy requirement while performing massive training exercises for such large and complex models, leading to severe environmental issues in the future (Boahen, 2022). Specifically, multiply and accumulate (MAC) operations in ANNs contribute ∼ 70–90% to the total operational cost (Jouppi et al., 2017). Therefore, developing energy-efficient neuromorphic hardware solutions has become one of the most critical challenges for future computing systems.

Hardware accelerators based on various types of nanoelectronic devices have already been proposed to improve performance of various neuromorphic applications and algorithms (Dutta et al., 2021; Dutta et al., 2020; Ge et al., 2018; Kumar and Bezugam, 2024; Oh et al., 2021; Oh et al., 2018; Park et al., 2024; Rafiq et al., 2023; Selcuk et al., 2024; Seo et al., 2020; Shi et al., 2018). For large and energy efficient neural network implementations, a synaptic device must show some of the main attributes, i.e., low energy consumption for read and write operations, scalability to achieve high synaptic density, high reliability, in-situ nonvolatile storage and computation, and linear and symmetric synaptic weight updates, i.e., upward and downward (Kuzum et al., 2012; Park et al., 2024). However, many synaptic device candidates still suffer from limited precision, large variations, and high energy consumption to achieve the required conductance values representing the weights of a network. There has been recent interest in exploring disordered physical systems which exhibit a complex energy landscape with a finite number of local minima exhibiting synaptic memory behavior. These have long been considered as models to describe emergent computational behavior displayed by neural networks including our brain from the perspective of statistical mechanics (Hopfield, 1982; Little, 1974; Niazi et al., 2024). Such systems ‘collectively’ host an almost continuum of states that can be used to represent synaptic memory configurations, in an alternative approach when compared to distributed synaptic memory states.

In this context, fluxon synapses based on superconducting loops combined with Josephson Junctions (JJs) arise as a promising technology that can offer several advantages, including low power consumption, high-speed operation, indefinitely large endurance and scalability.

JJ translate input excitations to flux flow (fluxons). JJs are the only switching elements in the system and superconducting loops are responsible for storage of trapped fluxon as circulating superconducting currents. These JJs can be switched without any degradation and cycled indefinitely without change (Duzer, 1989). It has also been validated that the switching current ‘I_C_’ of a well fabricated JJ is independent of repetition rate and applied magnetic field (or input) from one test to another (Schroen, 1968). Russek et al. (2016) proposed a fluxon based neuromorphic computing for large scale neuromorphic system with each JJ spiking at the rate of ~10^10^ per second. This indicates the indefinite reuse of fluxon based synaptic device if used under optimum operating conditions. These Josephson memory cells store information as persistent circulating currents and equivalent to fluxons in superconducting loops. No refreshing/rewriting is necessary because currents can be maintained indefinitely in ideal lossless superconducting loops (Zmpe, 1980). This method of storage is nonvolatile and has no power consumption after storing the state. In a superconducting loop system, the magnetic flux is quantized as fluxons (Φ_0_ = 2.065×10^−15^ T/m^2^), and cells have been built which operate either with only one flux quantum, or with many of them with non-destructive read (Wolf, 1978; Zmpe, 1980; Goteti et al., 2022). JJs work as bridges between superconducting loops and can operate at high speeds up to a few THz. JJs are interconnected due to macroscopic coherence with long-range interactions in superconductor loops and display a rich spectrum of memory states while having zero-static power dissipation (Goteti et al., 2022; Goteti and Dynes, 2021; Jué et al., 2022; Schneider et al., 2022). The memory states trapped in the form of fluxons in the loops result in stable flux flow pathways when excited with input signals. The flow pathways can be characterized as synaptic weights from statistical correlations of flux between Josephson Junctions.

Recently, a few studies have proposed disordered networks consisting of several superconducting loops with Josephson’s junction showing stable memory configurations of trapped fluxons in loops and movement of spike signals (e.g., neuronal activity) in small-scale disordered networks of superconducting loops (Goteti et al., 2022; Goteti et al., 2024; Goteti and Dynes, 2021). The study of physics of collective behavior of these randomly connected superconducting loops showed a great promise to perform some neuromorphic computations at small-scale (Goteti et al., 2022; Goteti and Dynes, 2021). However, to date such disordered superconducting loops have not been studied at the network-level to perform complex computations more relevant to modern AI models or to implement learning and inference tasks using a standard dataset. In this work, we explore crossbar architecture based on a previously studied configuration with three superconducting loops as individual synaptic elements and evaluate its performance using standard models and datasets. We experimentally characterize the fluxon synapses to investigate stable memory states when arranged into a crossbar structure to implement MVM operations in a 2-layer MLP neural network. We simulate fluxon synapse crossbar based synaptic core to perform learning and inference tasks on the MNIST dataset. Finally, we benchmark the performance and energy efficiency of the proposed superconducting (disordered) loop based synaptic device against other state-of-the-art synaptic device technologies.

## 2 Fabrication of Josephson Junction (JJ) and three superconducting loop device

A Josephson Junction (JJ) is a superconductor-insulator-superconductor (SIS) structure. A helium ion microscope can be used to selectively create a tunnel barrier (insulator region) in high-T_c_ (85 K) YBa_2_Cu_3_O_7_ (YBCO) superconductors to form a JJ by exposure to focused He^+^ ions. The detailed fabrication steps are provided in the methods section. A JJ generates quantized flux, also called fluxons when a current greater than a critical current I_C_ passes through the JJ (Fulton et al., 1973; Likharev and Semenov, 1991). A superconducting YBCO loop consisting of JJs can trap such quantized flux in the form of either clockwise or counterclockwise circulating supercurrents.

**Figure 1:**
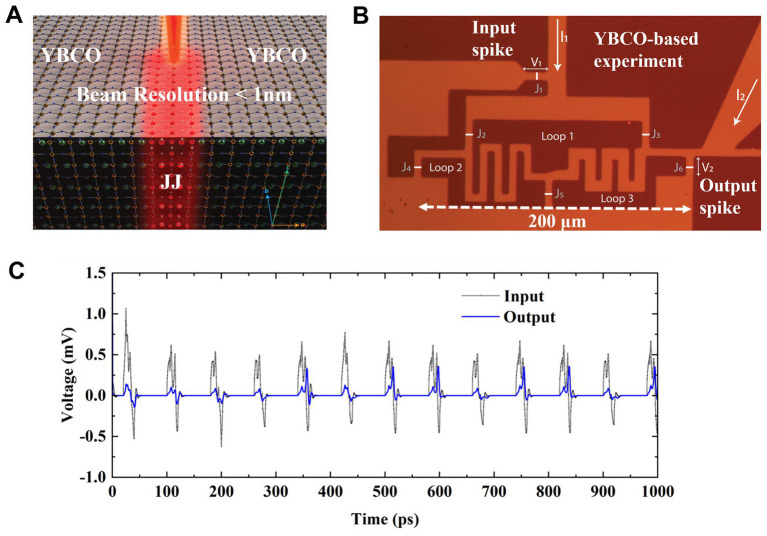
**(A)** Illustrates the fabrication of Josephson junction in between two YBCO superconducting regions. **(B)** Optical microscope image of a YBCO-based fabricated three superconducting loop circuit illustrating loops’ structure, all junctions from input (J1) to output junction (J6). All Josephson junctions lie within a square of a 200 μm x 200 μm of fabricated superconducting loop structure which is exposed to chemically remove the gold layer while maintaining the YBCO thin film. **(C)** Shows the high-frequency simulation of a few individual input spikes (fluxons) entering superconducting loops through input junction J1 and exiting through one of output-junction J6 (shown in **B**). At these timescales, the output spiking activity appears stochastic with a constant frequency activation at the input. Fluxon statistics is averaged over many spikes and results in a steady flux flow pattern and fixed output number.

A structure consisting of three YBCO superconducting loops has been fabricated with JJ tunnel barriers as shown in Figures 1A,B. The JJs connect loops together and allow fluxon movement in and out of the loops. The fluxons in the form of voltage spikes are fed in and out of the network of loops through JJs at input and output nodes as designated in Figure 1B. Loops labeled 1 to 3 can trap multiples of fluxons while an additional loop is used to induce individual fluxons through the input junction J_1_ using input current I_1_ as shown in Figure 1B. A constant flow of fluxons induced at the input can be measured by the voltage $V_{1}$ and the resulting output flow of fluxons can be measured as $V_{2}$ as illustrated in Figure 1B. Fluxons are injected at a frequency of $\frac{V}{\Phi_{0}}$ given by the Josephson Equations 1–3, where each individual fluxon is represented by a voltage spike and $\Phi_{0}$ represents a single fluxon as shown in Figure 1C.


(1) $$I=I_{c}\sin\varphi +\frac{\Phi_{0}}{2\pi R}\frac{d_{\varphi }}{dt}$$

(2) $$v=\frac{\Phi_{0}}{2\pi }\frac{d_{\varphi }}{dt}$$

(3) $$\int V\;dt=\Phi_{ο};\Phi_{0}=2.065\times 10^{-15};T/m2$$

Here, φ is the superconducting phase difference across the junction and $\Phi_{0}$ is the flux quantum.

**Figure 2:**
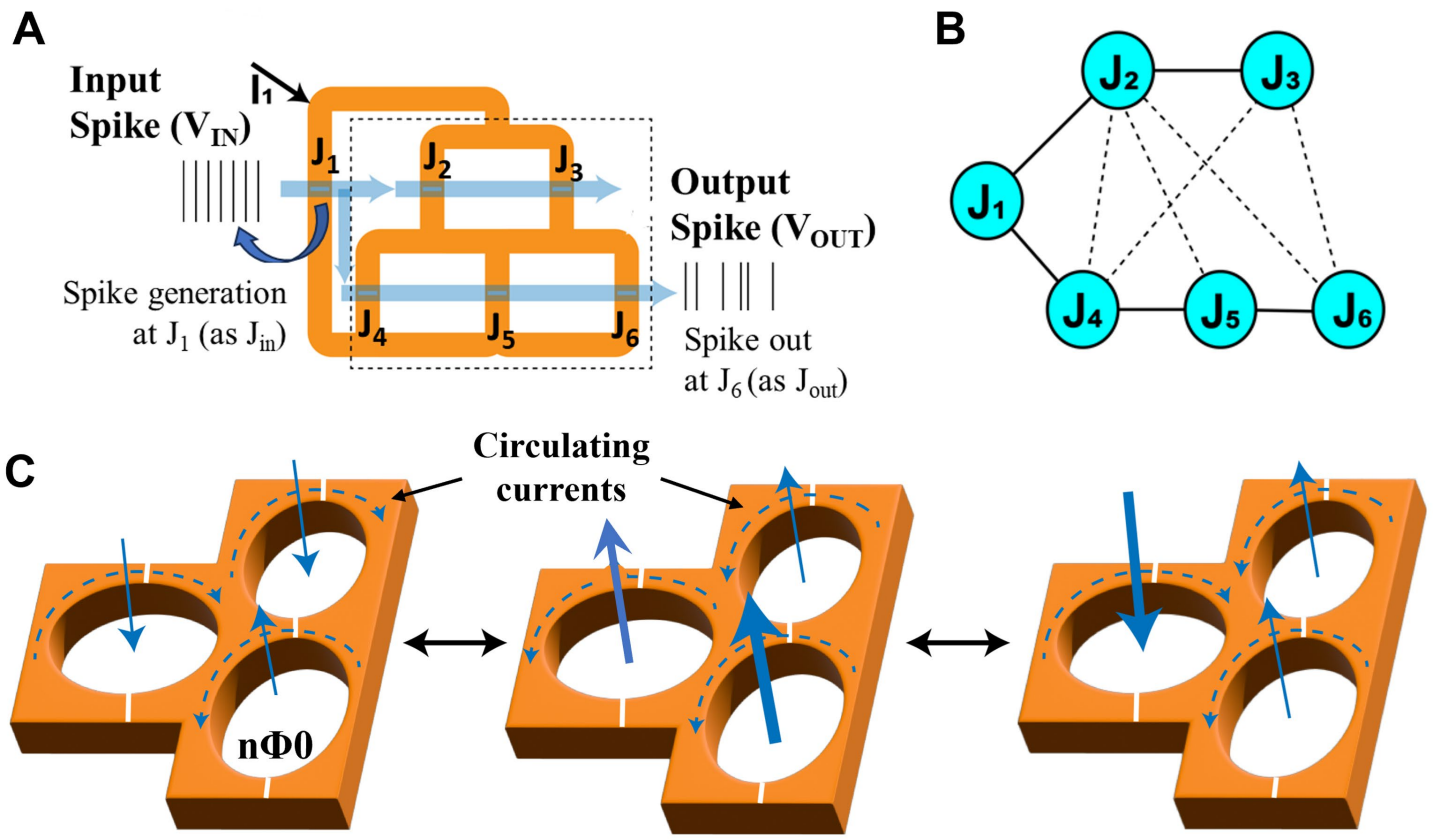
**(A)** Schematic of a fluxon synapse device based on three loop superconducting circuit with JJs. Spikes represented as equivalent voltages V_IN_ and V_OUT_ at J1 and J6. **(B)** A possible connectivity schematic of involved JJs in the superconducting loop network. **(C)** Schematics of superconducting loops with different fluxon configurations with circulating currents (clockwise and anticlockwise) representing non-volatile synaptic states.

A schematic of a simple three loop superconducting fluxon synapse circuit illustrating the loops, JJs, and input & output spikes is shown in Figure 2A. Input and output of fluxon synapses are spikes, represented as equivalent voltages ( $\overset{t}{\underset{0}{\int }}Vdt$ =nΦ_0_). When a constant flow of spikes (as V_IN_) is applied to the input (J1) of a three loop fluxon synapse, it produces a flow of spikes (as V_OUT_) at the output junction (J6) as illustrated in Figure 2A. The connected superconducting loops exhibit macroscopic coherence across all the connected loops. However, JJs can be strongly or weakly connected to each other, and the connection strength can be systematically programmed using the trapped fluxon configurations (Goteti et al., 2024; Goteti and Dynes, 2021).

Figure 2B shows this network level connectivity of JJs in the three superconducting loops consisting of 6 JJs. Two distinct signal flow pathways can be identified through J1-J4-J5-J6 and J1-J2-J3 in the superconducting structure as illustrated in Figure 2B. In this structure, fluxons can be stabilized into different flux configurations representing different nonvolatile synaptic weights as illustrated in Figure 2C. Each superconducting loop can trap multiples of fluxons (Φ_0_) and internally results in either clockwise or counterclockwise circulating supercurrents around each loops as illustrated in Figure 2C. The arrow width represents number (n) of trapped fluxons (Φ_0_) and direction represents a resultant circulating current in the loop either in clockwise or counterclockwise. A typical trapped fluxon configuration with circulating currents around an individual loops can be shown as nΦ_0_. The input flux diverges into these two pathways and one of these pathway signals can be read across the output node J6 as $V_{2}$ . The strength/weight of the pathway between input and output is defined as Equation 4.


(4) $$p=\frac{V_{2}}{V_{1}}=\frac{\#\mathrm{output fluxons}}{\#\mathrm{input fluxons}}$$

Several fluxon configurations are possible for this superconducting loop array, which result in the two flux (signal) flow pathways with different weights between input and output nodes. The amount of flux in individual loops together with the direction of the resulting input or output flux can be used to represent different weights. The output spikes individually mimic a stochastic pattern in time when observed at the pico-second scale as illustrated in Figure 1C using an exemplary high-frequency simulation of few individual input spikes/fluxons entering at input junction. However, when the spiking statistics are averaged for longer times (i.e., 500 ps) over many spikes it results in a steady flux flow pattern leading to stable synaptic weights between the pairs J1-J4, J4-J5, and J5-J6.

**Figure 3:**
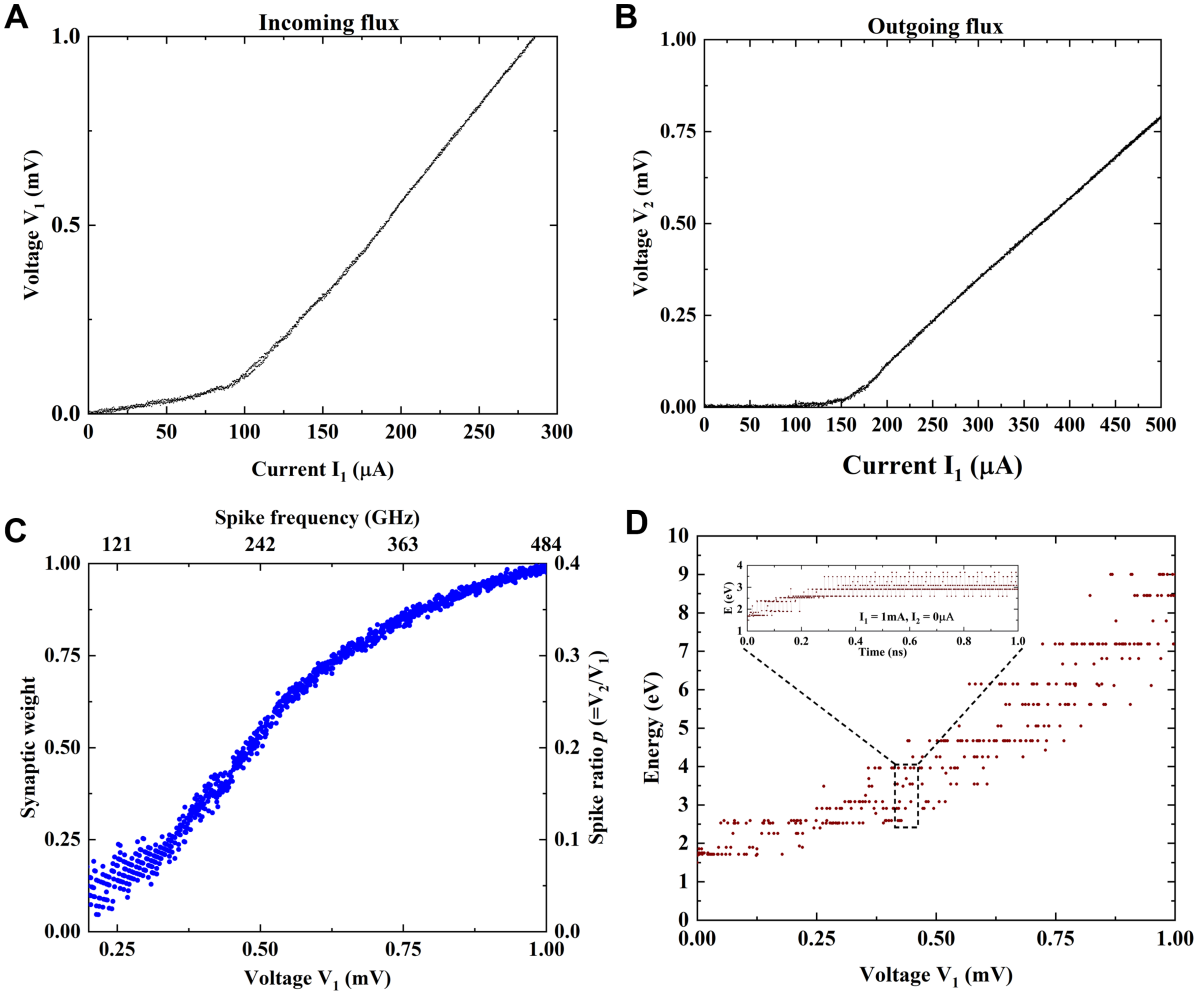
**(A)** Applied input current and measured voltage characteristics corresponding to fluxons (spikes) generated at input junction/node J1. **(B)** Applied input current and measured voltage characteristics corresponding to fluxons (spikes) generated at output junction J6. **(C)** Synaptic weights of different fluxon states as a function of input voltage and the corresponding input spiking frequency, derived from the I-V characteristics at zero bias current between input J1 and only selected output node J6. The calculated spiking ratio (using Equation 4) from 0 to 0.4 represents the connection weight between input and output. The calculated ratios are mapped to the synaptic weight from the lowest to the highest value. **(D)** Different states (denoted by their potential energy due to the trapped fluxons in the loops) achieved after relaxation as the input V_1_ flows systematically in the loops. Inset shows representative stable states.

The measured I-V characteristics at input (J1) and output (J6) junctions are shown in Figures 3A,B. The fluxon states as a function of voltage and the corresponding input spiking frequency derived from the I-V characteristics. Figure 3C contains the connection weights (fluxon states) between input J1 and only selected output node J6. Figure 3C shows a larger number of weights (ratioed value) due to a small input step size (~1uV) and larger sweep range up to 1 mV. However, adding an additional bias current (denoted by I_2_ in Figures 1B, 2A) enables accessing different energy minima (fluxon states) by reconfiguring the energy configuration space inside the three loops fluxon synapse device. The obtained synaptic weights of different fluxon states as a function of input voltage in Figure 3C corresponds to zero I_2_ bias current. With longer integration times the superconducting loops are subjected to steady flow input patterns and the resulting synaptic weights as a function of different input spiking frequencies is shown in Figure 3C. The fluxon flow rate at each junction is quantified by the number of discrete fluxons traversing the junction over a fixed period, which can be characterized by the constant average frequency or voltage. Adjusting the number of input fluxons within a fixed period can be interpreted as either a change in voltage (with fixed duration) or a change in fluxon frequency (with fixed amplitude). This relationship can be simply defined by considering the fluxons amplitude (Φ_0_) over a fixed integration time (i.e., f = V/(nΦ_0_)). Figure 3D shows the stable relaxed energy states when input $V_{1}$ is varied systematically from 0 to 1.0 mV. Figure 3D also shows the physical significance of fluxon storage in the superconducting loops as a change in synaptic strength. The energy of states in three loop circuit can be estimated corresponds to the excitation input voltage V_1_ (i.e., average flow rate ‘n’ is V_1_/Φ_0_) for a fixed duration of t_1_ = 1 ns. Discrete energy states get modulated using different pulse heights of input V_1_ as shown in Figure 3D. The value of the energy state is estimated using Equation (5) where, n is the number of input spikes, I_C_ – junction critical current (~100 μA), N - number of junctions between input and output, P_6_ – output spike ratio at J6, P_3_- output spike ratio at J3.


(5) $$\begin{array}{l} E_{\mathrm{3loop}}=n\;I_{C}\;\Phi_{0}+n\;I_{C}\;\Phi_{0}\;N_{J1-J6}\;\\ \;P_{6}+n\;I_{C}\;\Phi_{0}\;N_{J1-J3}\;P_{3} \end{array}$$

## 3 Superconducting loop array for neuromorphic computing tasks

**Figure 4:**
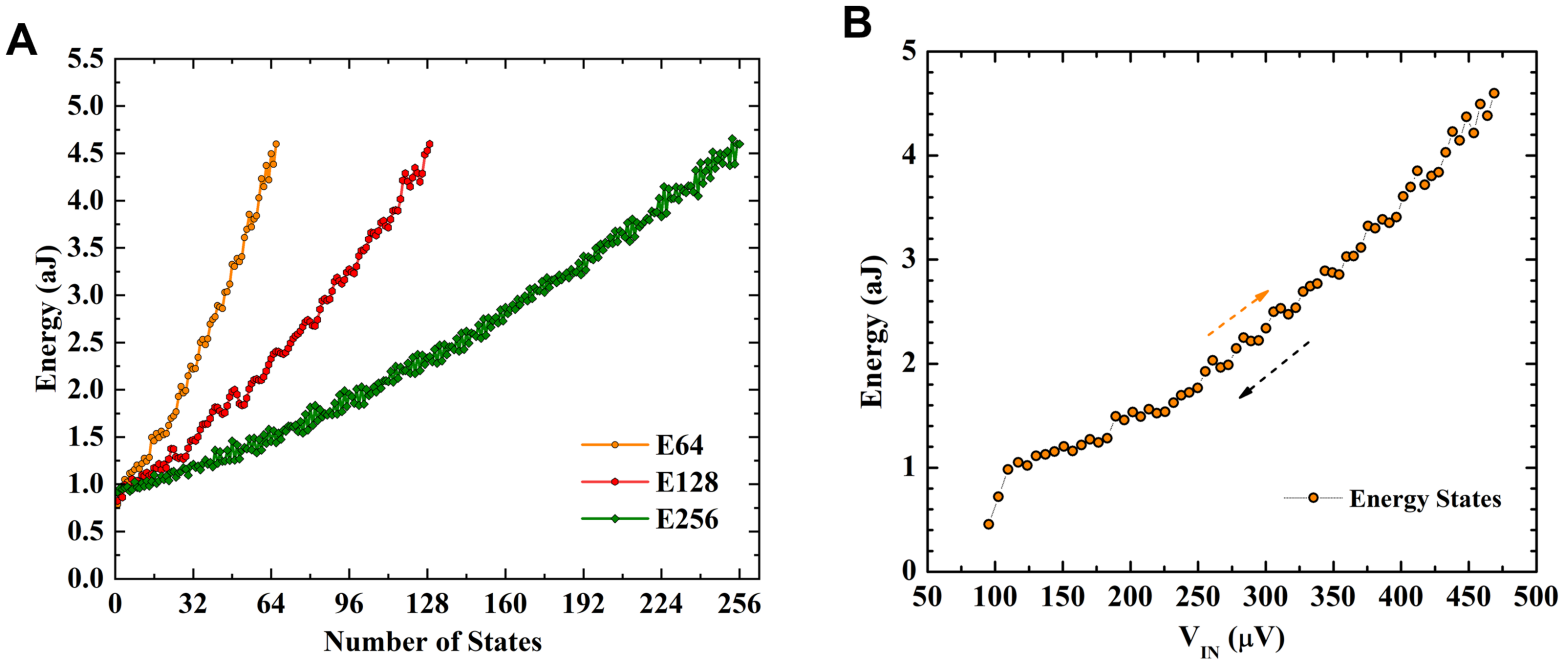
Number of energy states representing synaptic levels. **(A)** Different number of states (i.e., 64, 128, and 256) obtained using three superconducting loops under the controlled application of input fluxons. (as V_1_ in Figure 3) with different input steps. These different numbers of energy states correspond to input voltages varied in between ~100 μV and 500 μV with different step sizes (i.e., ~6 μV, 3 μV, and 1.5 μV). **(B)** Gradual modulation of energy states in both upward and downward directions with input voltages representing the number of applied input fluxons or rate of fluxons for the 64 states.

**Figure 5:**
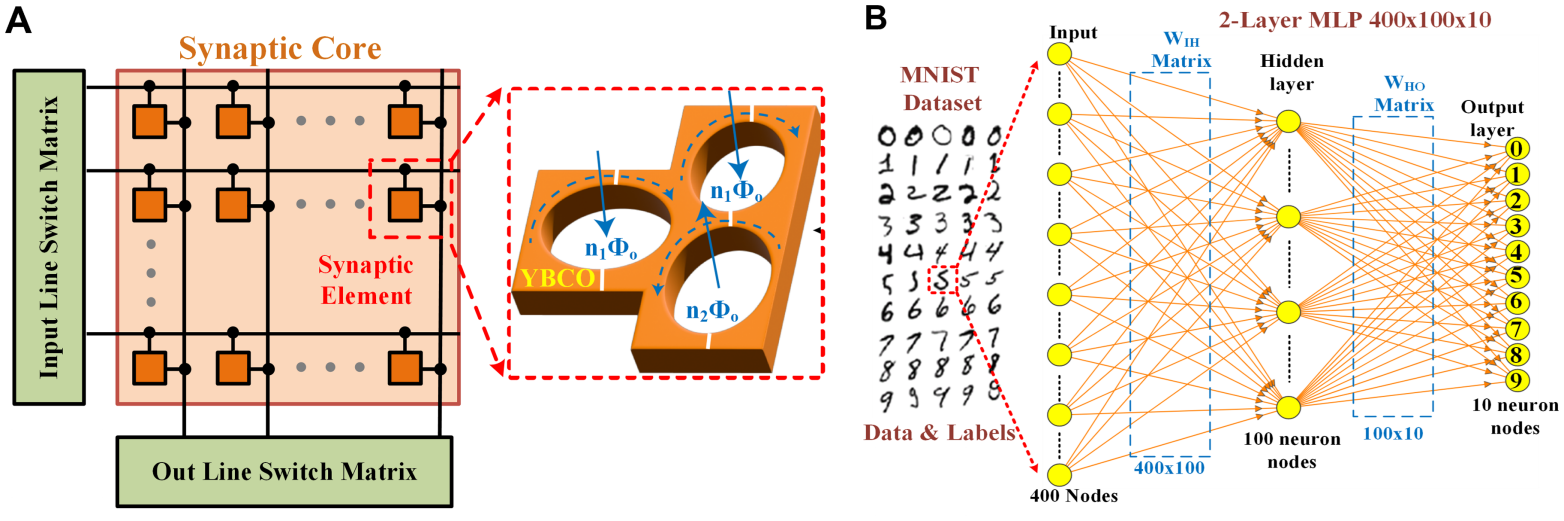
**(A)** Crossbar structure of the synaptic core implemented with three superconducting loops acting individual synaptic element. **(B)** Schematic of implemented MLP neural architecture and used MNIST Dataset for image classification.

Performing MVM operation in a crossbar structure is a widely used approach to accelerating neural networks towards achieving massive parallelism through in-memory computing (Gokmen and Vlasov, 2016; Hu et al., 2018; Li et al., 2018). In this section, we investigate crossbar arrays of fluxon synapses to perform MVM operations. Different numbers of synaptic weight states, i.e., 32, 64, 128, and 256 are obtained using fluxon synapses based on three superconducting loops in the form of energy states as shown in Figure 4A. These different numbers of states are achieved by varying input voltages in ~100 μV to 500 μV range with different step sizes (i.e., ~12 μV, 6 μV, 3 μV, and 1.5 μV). The write operation involves the application of high frequency spike input (equivalent to large write voltage) to reach the desired number of output fluxons (synaptic weight) whereas, the read operation involves application of a fixed lower frequency spike input (equivalent to smaller read voltage) to accumulate the output fluxon statistics without changing the internal energy state of the superconducting loops. The write operation excites the superconducting loops with a number of high frequency fluxons (e.g., 100 GHz) generated by a higher input voltage (≥100 μV) to induce a change in the energy state. Synaptic weight is modulated by changing the fluxon excitation frequencies (or equivalent input voltages, Figure 3C). During read operations, we use the low frequency fluxons (e.g., 1 GHz) generated by a smaller input voltage (2.065 μV). Therefore, for the MVM implementation with crossbars utilize low fluxon excitation frequency (~1 GHz), which could be understood as a low amplitude read signal that will not disturb the programmed weights. Figure 4B shows upward and downward changes in energy states during implementation of the nonvolatile synaptic weight update operation in a neural network with input voltage step size change of 6 μV to obtain 64 states. In the fluxon synapse device, there is no distinction between LTP and LTD states. When the device is properly configured, a particular synaptic state (energy minima) corresponds directly to the absolute input signal, regardless of LTP or LTD operation. These nonvolatile changes in the internal energy states represent weight-matrix elements in the synaptic core as shown in Figure 5A. In the system-level simulation framework, we employed an incremental/decremental pulse scheme with identical specifications to achieve the different states in the synaptic core.

### 3.1 Neural network implementation and system-level performance benchmarking

The fluxon synapse device can be used to store and update/train the weights of multilayer perceptron (MLP) neural network implementation. We performed system level assessment using experimental data from fluxon synapse devices for classification of the handwritten digits from MNIST dataset (LeCun, 1998) consisting of 60,000 training and 10,000 test images. The implemented 2-layer MLP network of sized 400(input)-100(hidden)-10(output) nodes, where the edge cropped 20×20 MNIST images are used for 400 input nodes as illustrated in Figure 5B. Two different sizes of weight matrices, W_IH_ (40,000) and W_HO_ (1,000), corresponding to input-hidden and hidden-output layers respectively, are implemented (Figure 5B).

To evaluate the system level hardware performance for online-learning, we used the circuit-level macro-model NeuroSim platform (Chen et al., 2018). We emulated the synaptic core hardware for MLP network using the fluxon synaptic devices. For benchmarking purposes, we performed the online learning within NeuroSim framework for fluxon synaptic core and various other synaptic devices. For emulating the proper crossbar level operation, we assumed superconducting interconnections with extremely low resistivity. These interconnects are used inside the crossbar architecture to connect fluxon synapses and propagate the required signals in and out of the array. For benchmarking, we focused on the metrics for the crossbar arrays and did not include the periphery for a fair comparison between different synaptic device technologies.

**Figure 6:**
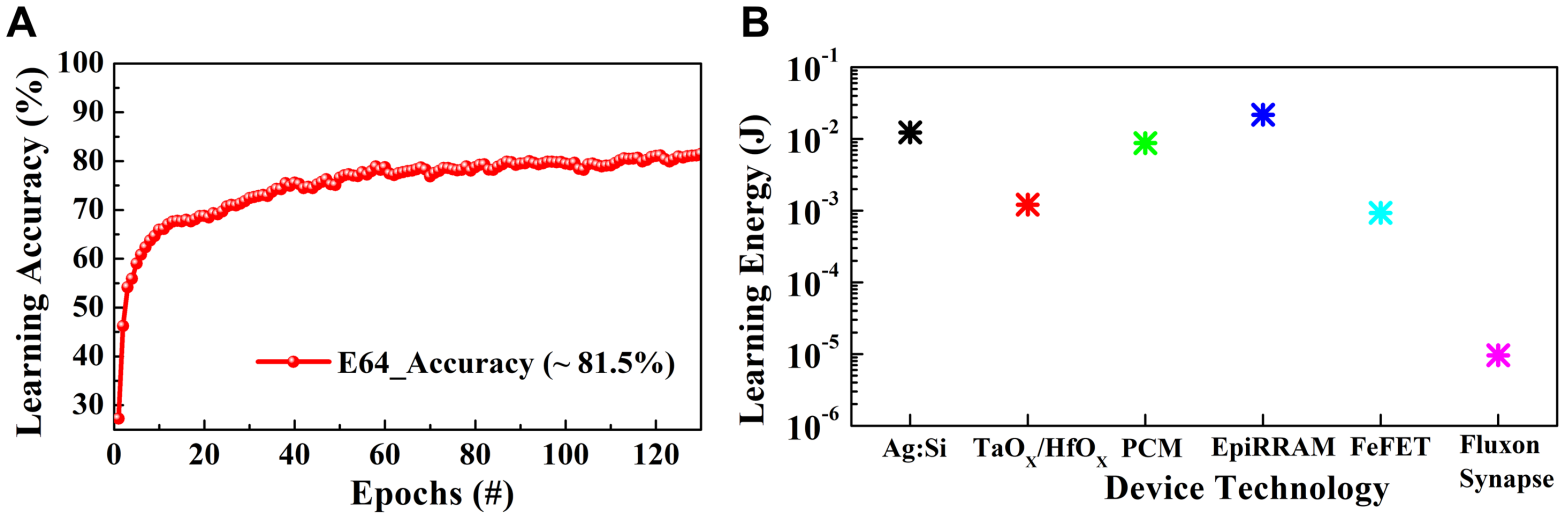
**(A)** On-chip learning accuracy of implemented MLP network for MNIST image classification over 125 epochs with 64 synaptic states using the fluxon synaptic core. **(B)** Online learning energy consumption of different synaptic cores using MNIST dataset over 125 epochs.

For on-line learning with the MLP network, we used low precision step activation function for simpler hardware implementation and Adam optimizer. We utilized backpropagation as the weight update algorithm. The network is trained over 125 epochs with Adam optimizer to obtain classification accuracy results using 64 synaptic/energy states per crosspoint, as shown in Figure 6A. In case of high C2C variations Adam optimizer with lower learning rates compared to in case of stochastic gradient dissent (SGD) optimizer shows better accuracy with an increase in energy consumption. SGD shows relatively higher variance in results and low accuracy due to stochastic gradient choice between application of gradient function while minimizing the training error. However, it converges faster than other optimizers and provides the optimal solution in case of large number of training/test cases with small C2C variations. SGD also has smaller memory footprints and learns quickly (Gupta et al., 2021; Ruder, 2016; Zhou et al., 2020). Finally, we benchmarked the fluxon synaptic core implementation with different synaptic crossbars using other state of the art device technologies, i.e., RRAM, PCM, FeFET etc. The fluxon synaptic core reduces the overall on-chip learning energy requirement significantly while achieving comparable learning accuracy using only 64 synaptic states as shown in Figure 6B and Table 1.

**Table 1:** System level benchmark results for state-of-art synaptic devices.

Devices	Ag:a-Si (Jo et al., 2010)	TaO_X_/HfO_X_ (Wu et al., 2018)	GST-PCM (Kuzum et al., 2012)	EpiRAM (Choi et al., 2018)	FeFET (Jerry et al., 2017)	Fluxon Synapse Device (This work)
Synaptic States	97	128	100	64	32	64
Weight update NLs	2.4, −4.88	0.04, −0.63	0.105, 2.4	0.5, −0.5	1.75, 1.46	−1.25
ON/OFF	12.5	10	19.8	50.2	45	~10.5
Weight increase pulse	3.2 V, 100 μs	1.6 V, 50 ns	0.7 V (avg.), 6 μs	5 V, 5 μs	3.65 V (avg.), 7 ns	382 μV (avg.), 0.5 ns
Weight decrease pulse	−2.8 V, 300 μs	1.6 V, 50 ns	3 V (avg.), 125 ns	-3 V, 5 μs	−2.95 V (avg.), 75 ns	382 μV (avg.), 0.5 ns
C2C variation	3.5%	3.7%	1.5%	2%	0.5%	9%
Online learning accuracy	~ 72%	~ 80%	~ 89%	~ 92%	~ 88%	~ 81.5%
Synaptic array energy	12.3 mJ	1.21 mJ	8.75 mJ	21.7 mJ	0.93 mJ	9.6 μJ

## 4 Discussion

In this work, we investigated and evaluated potential performance of a synaptic core made of fluxon synapse devices for highly energy efficient neuromorphic computing. The fluxon synapse device exhibits nonvolatile states as well as gradual modulation of states by the application of varied fluxon excitation frequency. Our experimental results show that the fluxon synaptic devices have the capability to provide a significantly large number of synaptic states, which can be leveraged for implementation of low energy on-chip learning with high precision weights. We performed system-level simulations for a hardware implementation of MLP network with the fluxon synaptic core. We benchmarked the superconducting loop synaptic core against the state-of-art synaptic devices, i.e., RRAM, PCM, EpiRAM, and FeFET. Our results suggest up to ~100x potential improvement in energy consumption for online learning over other technologies. It is important to mention that our analysis currently does not involve the cooling costs, which are difficult to estimate. Today, cooling costs are no longer specific to low temperature computing systems such as quantum computers or cryogenic CMOS. Data centers invest more than a third of their power budget on cooling costs, the aim of which is simply to prevent servers from shutting down induced by overheating (Saligram et al., 2024). Further work is needed for an accurate assessment of cooling costs for all new technologies. In addition, there is an increasing number of application areas for specialized low temperature computing, including cryogenic CMOS (Saligram et al., 2024) and quantum computing (Riel, 2021). Hardware accelerators based on fluxon synaptic arrays can be operated at liquid nitrogen temperature (~77 K) (Murduck, 2001). Parallel research and development of quantum computing and cryogenic CMOS may result in cheaper cooling, and it may provide a new and unique application for the superconducting loop devices for high performance AI applications.

## Data Availability

The original contributions presented in the study are included in the article/supplementary material, further inquiries can be directed to the corresponding author.
