# Supplementary material for: Evaluation of fluxon synapse device based on superconducting loops for energy efficient neuromorphic computing
Source: Front Neurosci. 2025 Feb 14;19:1511371. doi: 10.3389/fnins.2025.1511371 (PMC11868091; doi:10.3389/fnins.2025.1511371)
Supplement: Supplementary file 1 [file Data_Sheet_1.pdf]

Supporting Information

Evaluation of Fluxon Synapse Device Based on Superconducting Loops for Energy Efficient Neuromorphic Computing

Ashwani Kumar[1], Uday S. Goteti[2], Ertugrul Cubukcu[3], Robert C. Dynes[2], and Duygu Kuzum[1]

[1]Department of Electrical and Computer Engineering, University of California San Diego, CA, USA. [2]Department of Physics, University of California San Diego, CA, USA. [3]Department of Chemical and Nano Engineering, University of California San Diego, CA, USA.

Methods

Learning Energy Calculation for Fluxon Synaptic Core

In the synaptic core, energy consumption is primarily due to static energy (i.e., current flow through the synaptic cells). The energy consumption of a selected fluxon synapse cell during the weight increase/decrease phase is given by the following equation s1.

$$E_{CELL} = V_{in}\times I_{CELL}\times N_P\times T_P \qquad \text{(s1)}$$

V_{in} is the input weight read/write voltage. I_{CELL} is the total current following through the selected synaptic cell. N_P is the number of applied pulses and T_P is the pulse width.

Beyond the synaptic cell, energy consumption also accounts for the resistivity of core/array wires. In our case, this contribution is negligible due to the superconducting nature of the synaptic core. Finally, the total energy consumption of the synaptic core is estimated by summing the energy consumption for all read and update operations performed during training for 125 epochs.

Fabrication of Fluxon Synapse Device

To reduce nonuniformity from the starting material, we used commercially available $YBa_2Cu_3O_7$ (YBCO) films (Ceraco) which were thermally co-evaporated on sapphire wafers and buffered by cerium oxide (CeO_2). These 35nm thick YBCO films with 200nm gold capped show high critical temperature ($T_c \geq 85K$), and low critical current density variation across the whole wafer. Such high Tc superconductors (e.g., YBCO) undergo a superconductor to insulator transition if a point defect disorder is created by high energy ion damage. This property of YBCO is used to fabricate planar thin film Josephson junctions (JJ). The superconducting loop's structure consists of bulk electrodes, ground plane, and in/out terminals are defined using photolithography and ion milling process. A focused 0.5pA He+-ion beam accelerated at 32.5 kV from helium ion microscope induced tunnel barriers for JJs. The 825-photoresist has been developed with Fuji OCG-934 for 45s at 5000 rpm. A Microtech laser writer exposed the photoresist with a 405-nm GaN solid-state laser defining the layout pattern. After that it is mounted into a broad-beam

argon ion mill. This ion milling isolated the traces and loops of the layout design by milling away the material. A second lithographic step was performed to open apertures in the gold capping layer such that the helium ion irradiation could be incident directly on the YBCO layer. Targeted locations for JJs are exposed to Ki+ etch to chemically remove the only gold layer. An optical image of the final output of these fabrication steps as a 200μm*200μm fabricated structure is presented in Figure 1(b). However, the focused ion beam (produced in the NanoFab) can be focused to a beam spot size on the scale of 1 nm and be controlled within nanometer resolution. This beam was rastered in a line across the lithographically defined electrodes, introducing an average ion fluence of 4 $\times 10^{16}$ ions/nm to define the Josephson barriers. Ion fluence influences the nature of the barrier and effects the critical current of the Josephson junctions.

Characterization Setup

In our current test setup, we used the liquid helium storage dewar at ~28K. The fabricated samples were mounted on a J-lead chip carrier using Al wire bonding for electrical measurements. The chip carrier was inserted in dewar using a cryogenic insert probe backfilled with 500 mtorr of helium gas for temperature exchange. After colling, the temperature was controlled by adjusting the tip height in relation to the liquid helium surface inside the dewar. All experimental results presented for three superconducting loops were performed at ~28K in a liquid helium storage dewar using a cryogenic insert probe.

Fluxon Synapse Model

We implemented an equivalent lumped element circuit model of the fluxon synapse device based on three superconducting loops network with JJ, and inductors, etc. The resistive and capacitive shunted (RCSJ) model is used for JJs. The loop sizes are designed to accommodate several trapped fluxons (i.e., $LI_C/\Phi o > 1$) by choosing appropriate values of inductances (L) and critical currents (I_C). The stable flux states in superconducting loops are perturbed by any applied excitation during both the read and the write processes. However, the flux flow pathways measured over the integration time period is stable while the underlying memory state is only perturbed within its vicinity.

While the write process involves applying the required large excitation currents which are active for a longer time than the integration period required to reach a desired flow or the synaptic weight, read process involves weakly perturbing the state with smaller excitations for a long period to accumulate the flux flow statistics. Therefore, we have considered a fixed number of fluxons (i.e., SFQ spikes) of 100 for both "read" and "write" operations. However, the write operation involves exciting the network with 100 fluxons over a short period (e.g., 1THz) to induce a memory state change, while the read operation involves the same 100 fluxons but over a long period (e.g., 1GHz). The exact excitation flux frequencies will be different for different memory states, but the average values of 1THz and 1GHz over 100 fluxons are assumed for all the states to achieve a reasonable estimate for the energy efficiency calculations.

System-level Benchmarking Simulations

“We simulated online learning accuracy and circuit-level performance for the designed synaptic core architecture using NeuroSim. At first, we extracted the parameters for the superconducting loops such as number of synaptic states, states/weights update non-linearity, state change dynamic range (max/min ratio), state read & write voltages from experimental characteristics. We simulated crossbar arrays based on superconducting loops for weighted sum and weight update operations as shown in Figure 5(a).

To simulate crossbar type architecture for fluxon synapses, we used experimentally measured resistivity of the superconducting interconnects. For simulations, we used the binary activation function and different bit precisions (i.e., 6, 7, and 8-bits) for network weights representing positive weight values (W=0~1). The input vector is encoded using either read or write voltage pulse amplitudes & widths for weighted sum and weight update operations, respectively. During online learning using fluxon synapse synaptic core, images were randomly picked from the MNIST training dataset for 125 epochs with 8000 images per epoch which leads to the training of the MLP network using back-propagation algorithm for a total 1 million images. For fair comparison and benchmarking, we kept the same periphery architecture at 32nm tech node for all simulations and excluded the peripheral circuits results circuits at the end.”
